# A walking programme and a supervised exercise class versus usual physiotherapy for chronic low back pain: a single-blinded randomised controlled trial. (The Supervised Walking In comparison to Fitness Training for Back Pain (SWIFT) Trial)

**DOI:** 10.1186/1471-2474-10-79

**Published:** 2009-07-02

**Authors:** Deirdre A Hurley, Grainne O'Donoghue, Mark A Tully, Jennifer Klaber Moffett, Willem van Mechelen, Leslie Daly, Colin AG Boreham, Suzanne M McDonough

**Affiliations:** 1 School of Physiotherapy & Performance Science, University College Dublin, Dublin 4, Ireland; 2 Centre of Excellence for Public Health (Northern Ireland), Queens University Belfast, Mulhouse, Royal Victoria Hospital, Grosvenor Road, Belfast, BT12 6BJ, Northern Ireland; 3 Institute of Rehabilitation, University of Hull, 215 Analaby Road, Hull, HU3 2PG, UK; 4 EMGO Institute and Department of Public Health and Occupational Health, VU University Medical Center, Amsterdam, The Netherlands; 5 School of Public Health and Population Science, University College Dublin, Dublin 4, Ireland; 6 Institute for Sport and Health, Phillips Building, University College Dublin, Dublin 4, Ireland; 7 Health and Rehabilitation Sciences Research Institute, University of Ulster, Jordanstown Campus, Antrim, BT37 0QB, Northern Ireland

## Abstract

**Background:**

Chronic low back pain (CLBP) is a persistent disabling condition with rising significant healthcare, social and economic costs. Current research supports the use of exercise-based treatment approaches that encourage people with CLBP to assume a physically active role in their recovery. While international clinical guidelines and systematic reviews for CLBP support supervised group exercise as an attractive first-line option for treating large numbers of CLBP patients at low cost, barriers to their delivery include space and time restrictions in healthcare settings and poor patient attendance. The European Clinical Guidelines have identified the need for research in the use of brief/minimal contact self-activation interventions that encourage participation in physical activity for CLBP. Walking may be an ideally suited form of individualized exercise prescription as it is easy to do, requires no special skills or facilities, and is achievable by virtually all ages with little risk of injury, but its effectiveness for LBP is unproven.

**Methods and design:**

This study will be an assessor-blinded randomized controlled trial that will investigate the difference in clinical effectiveness and costs of an individualized walking programme and a supervised general exercise programme compared to usual physiotherapy, which will act as the control group, in people with chronic low back pain. A sample of 246 patients will be recruited in Dublin, Ireland through acute general hospital outpatient physiotherapy departments that provide treatment for people with CLBP. Patients will be randomly allocated to one of the three groups in a concealed manner. The main outcomes will be functional disability, pain, quality of life, fear avoidance, back beliefs, physical activity, satisfaction and costs, which will be evaluated at baseline, and 3, 6 and 12 months [follow-up by pre-paid postage]. Qualitative telephone interviews and focus groups will be embedded in the research design to obtain feedback about participants' experiences of the interventions and trial participation, and to inform interpretation of the quantitative data. Planned analysis will be by intention to treat (quantitative data) and thematic analysis (qualitative data)

**Discussion:**

The trial will evaluate the effectiveness of a walking programme and a supervised general exercise programme compared to usual physiotherapy in people with CLBP.

**Trial registration:**

Current controlled trial ISRCTN17592092

## Background

Chronic low back pain (CLBP) is a persistent disabling condition with rising significant healthcare, social and economic costs [1,2]. Current research and both European and American Clinical Guidelines supports the use of exercise-based treatment approaches that encourage people with chronic low back pain (pain >3 months) to assume a physically active role in their recovery [3-6]. However, these patients often report decreased habitual physical activity levels, believing that if movement hurts they may be re-injuring themselves, termed 'fear avoidance'[7,8].

The recent European Clinical Guidelines for CLBP concluded that supervised group exercise is an attractive first-line option for treating large numbers of CLBP patients at low cost [6]. The "Back to Fitness" physiotherapy-led supervised group exercise programme for CLBP was introduced in the UK in the 1990s [9]. Its effectiveness has been supported in several RCTs, reporting significant improvements in pain and disability compared to 'routine' physiotherapy (i.e. advice/education, passive mobilisation/manipulation)[10] and GP management [11], and it has been shown to be cost effective[11]. Nonetheless, a national survey by the Principal Investigator of public general hospitals in the Republic of Ireland (ROI), found that only 39% of responding physiotherapy departments were delivering group-based exercise programmes for CLBP, the main barriers being space and time restrictions, and insufficient staffing levels [12].

Furthermore, another limiting factor from the patients' perspective is poor adherence with the recommended exercises [13], and the requirement for regular attendance at the class with drop out rates of up to 30% being reported in the literature [14]. Given the difficulties and limited availability of supervised exercise programmes, an alternative clinically and cost effective approach to increasing the activity levels of patients with CLBP is warranted.

The European Clinical Guidelines have identified the use of brief/minimal contact self-activation interventions that encourage participation in physical activity for CLBP as an area for future research, particularly as this approach could result in significant cost savings if it proves to be at least as effective as other treatments [6]. For CLBP, there is moderate evidence from RCTs [15,16] and a systematic review [3] that brief information and advice to stay active are more effective than usual GP care in reducing LBP-related disability, but not pain levels. However, there is limited evidence of the effects of self-activation interventions compared to supervised exercise programmes on pain and disability levels [17], and no evidence of the effects of either type of programme in increasing CLBP patients' level of participation in physical activities, return to work rates or psychosocial variables compared to 'routine' physiotherapy.

Walking may be an ideally suited form of exercise prescription as it is easy to do, requires no special skills or facilities, and is achievable by virtually all ages with little risk of injury [18,19]. General physical activity recommendations encourage individuals to accumulate 30 minutes of moderate intensity physical activity on five days per week [20] or 10,000 steps [21]; and there is evidence from a meta-analysis that healthy but sedentary individuals who take up a programme of regular brisk walking improve several known risk factors for cardiovascular disease [22]. There is limited evidence that people with LBP are less physically active and have altered patterns of physical activity than matched controls [23,24]. Several recent studies have reported that people with chronic LBP failed to reach the recommended 10,000 steps per day and took significantly less steps than healthy age-matched controls [24,25].

There has been minimal investigation of the effectiveness of walking programmes in LBP management. One RCT found unsupervised walking at a self-selected pace was less effective than individual physiotherapy or medical exercise therapy in reducing pain, disability, costs and increasing patient satisfaction levels in people with CLBP [26]. Participants in the walking group were instructed to walk for one hour three times per week at their convenience, preferably on alternate days. However, apart from one information session, participants in the walking group received no further contact or support from a health professional, and their level of compliance or physical activity was not investigated. From a North American study back pain sufferers who participated in brisk walking for at least 3 hours per week reported reductions in low back pain, disability, and psychological distress [27]. Nonetheless, that trial was primarily designed to compare chiropractic and medical care for LBP, and thus patients did not receive any structured physical activity or exercise interventions and no details of the walking intervention are provided. Given the limited research to support or refute walking programmes in CLBP management, there is a need to investigate the effectiveness of such programmes in people with chronic LBP.

For the researchers designing such walking programmes, and the clinicians delivering them, the challenge is to devise an effective intervention that will motivate habitually sedentary people with LBP to be physically active. A Cochrane systematic review of interventions to promote physical activity concluded that a mixture of self-direction and on-going professional support can encourage adults to be more physically active [28]. A randomised controlled trial found that an eight-week low frequency progressive walking programme for 'normal' sedentary adults had high levels of adherence, and resulted in increased physical activity levels with no adverse effects or injuries, thus increasing its clinical application [29]. Another trial reported evidence for the benefit to fitness and cardiovascular risk of the '30 minute brisk walking, five days per week' message to people aged 50–65 years who participated in a 12 week unsupervised home-based walking programme that used pedometers as motivational tools [30]. Pedometers are simple to use, inexpensive devices that produce a user-friendly output (step count, duration) that serves as an effective motivator to increase physical activity [31].

A self-activation programme by walking may be a more pragmatic and less costly intervention than supervised exercise classes to promote increased physical activity levels in patients with CLBP. It has the additional advantages of supporting a larger population of CLBP patients at the same time, and by moving the emphasis away from the hospital setting could reduce the costs to the Health Service. Given that the majority of patients with CLBP currently receive a range of interventions within the scope of 'usual' physiotherapy (advice, passive mobilization/manipulation, general exercise [32-34], this RCT will establish the difference in effectiveness of a walking programme and a supervised general exercise programme compared to usual physiotherapy, which will act as the control intervention, in subjects with chronic low back pain.

The *primary objective *is to:

(i) determine the difference between groups in mean change in functional disability at 6 months

The *secondary objectives *are to:

(ii) determine the difference between groups in mean changes in pain, health-related quality of life, psychosocial beliefs, days of sick leave, daily physical activity levels, self efficacy, readiness to change and patient satisfaction

(iii) determine the difference between groups in cost utility and the cost effectiveness of the alternative treatment programmes

(iv) determine the difference between groups in level of adherence to each intervention

(v) complete a qualitative exploration of subjects experience of each intervention

### Hypotheses Tested

The trial will test two 'null' hypotheses. The primary null hypotheses is that,

(a). in people referred for physiotherapy for chronic LBP there is no difference in clinical outcome and costs between those receiving a walking programme, a supervised general exercise programme and usual physiotherapy;

The secondary null hypothesis is that:

(b). subjects that have received a walking programme or supervised general exercise programme will not have changed daily physical activity levels compared to those who have received usual physiotherapy

## Methods/design

The Research Ethics Committees of the participating Dublin hospitals have granted approval for this study:

1. Adelaide and Meath Hospital incorporating the National Children's Hospital

2. Beaumont Hospital

3. Connolly Hospital

4. Mater Misericordiae University Hospital

5. St Vincent's University Hospital

**Figure 1:**
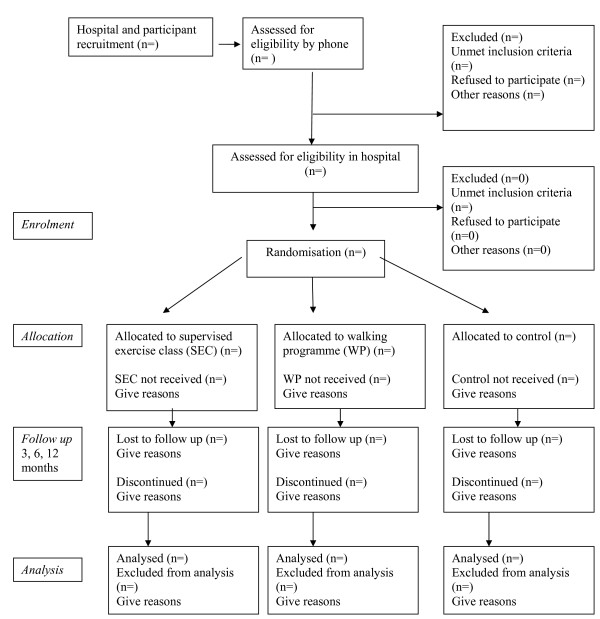
**Participant flow through the RCT (based on CONSORT statement)**.

The trial will be reported according to the recommendations of the CONSORT statement [35] and the flow of participants through the study is represented in Figure 1. The quantitative study will establish the difference in clinical effectiveness and costs of a walking programme and a supervised general exercise programme compared to usual physiotherapy, which will act as the control intervention, for patients with chronic low back pain. The qualitative study will explore participants' experience of the study and the interventions.

### Quantitative study

#### Design

The study will be a prospective randomized controlled trial (RCT) with three arms (i) walking programme, (ii) a supervised exercise class, (iii) usual physiotherapy of up to 8 weeks duration. Outcomes will be assessed at baseline and 3, 6 and 12 months [follow-up by pre-paid postage].

#### Controlling bias

The RCT design includes key methodological features that have been recognized as important in minimizing bias in clinical trials: true randomization, concealed allocation, specification of eligibility criteria, blind outcome assessment, blind analysis and intention-to-treat analysis.

#### Setting

A sample of patients will be recruited in Dublin, Ireland through the physiotherapy departments of acute public hospitals that provide physiotherapy treatment for people with CLBP.

#### Protocol protection

The following mechanisms will be used to ensure the trial protocol is applied consistently: protocol manuals will be developed and all involved researchers and clinicians will be trained to ensure that subject screening, assessment, random allocation and treatment procedures are conducted according to the protocol; a random sample of 20% of treatment records in each group will be audited by a researcher not involved in the day to day running of the trial to check that treatment is administered as per the protocol; if any anomalies are found all treatment records will be checked. The treatment record forms will be completed by clinicians on every patient recruited to the study.

### Study population and recruitment

#### Clinics and Clinicians

The study will be conducted in several Dublin metropolitan acute general hospitals physiotherapy departments that provide rehabilitation for musculoskeletal conditions. The relevant hospitals will be contacted by telephone and email seeking expressions of interest to participate in the study. A meeting will be scheduled with each hospital's physiotherapy department manager to outline the project and distribute the outline protocol. Information meetings will be arranged with managers and treating physiotherapists to discuss the study background, aims and methodologies and to address their queries. Reception staff will be briefed on the study recruitment process, and patient appointment systems, paperwork and filing arrangements will be made. All participating physiotherapists involved in the interventions will attend training days in the School of Physiotherapy and Performance Science, University College Dublin. These days will be delivered by various members of the research team. The main focus of the training days will be to ensure that the interventions are standardized across all centres. A cognitive behavioural therapy (CBT) approach will be emphasized and performance indicators will be set for the physiotherapists to further ensure homogeneity of the active interventions.

In advance of the training days, a detailed trial manual will be distributed to the therapists. Their role in the study will be highlighted. They will also complete the PABs PT (Pain Attitudes and Beliefs of Physiotherapists) Questionnaire to establish their biopsycho-social or biomedical orientation to the management of CLBP [36].

#### Patients

All eligible patients with chronic or recurrent LBP referred to the participating hospitals physiotherapy departments by general practitioners or hospital consultants will be invited to participate in the study by the Trial Co-ordinator in order of referral until adequate subject numbers are achieved. Patients will receive the initial contact by telephone to explain the trial procedures, to clarify interest and to screen eligibility, and will then be followed up by a letter containing the patient information sheet and an invitation to attend for baseline assessment. Suitable, interested patients will attend the relevant hospital's physiotherapy department, where detailed verbal explanations of the study protocol will be provided and, written informed consent will be sought by the Trial Co-ordinator. The Trial Co-ordinator will record the number of patients invited, the number who declined, ineligible patients and reasons.

### Eligibility Assessment

#### Clinicians

Chartered Physiotherapists who are eligible for membership of the Irish Society of Chartered Physiotherapists and are employed by one of the participating hospitals are eligible to participate.

#### Patients

At the initial telephone contact and the baseline interview the Trial Co-ordinator will use a screening checklist to verify eligibility (Table 1). The Physical Activity Readiness Questionnaire (PAR-Q)[37] will be completed to determine whether medical clearance is necessary before trial participation.

**Table 1:** Eligibility Criteria for the SWIFT Trial

*Inclusion criteria*	*Exclusion criteria*
Patients with chronic (≥3 months) or recurrent (≥3 episodes in previous 12 months) LBP of mechanical origin with/without radiation to the lower limb	Currently or having received treatment for CLBP within previous 3 months

Males/females between 18–65 years	Red flags indicating serious spinal pathology, e.g. cancer, cauda equina lesion

No spinal surgery within the previous 12 months	Radicular pain indicative of nerve root compression

Patients deemed suitable by their GP/hospital consultant to carry out an exercise programme	Patients diagnosed with severe spinal stenosis, spondylolisthesis, fibromyalgia

Patients willing to attend for an 8-week treatment programme of exercise classes	History of systemic/inflammatory disease, e.g. rheumatoid arthritis

Access to a telephone (for follow-up support)	Patients with any confounding conditions such as a neurological disorder or currently receiving treatment for cancer

Fluency in English (verbal and written)	Patients with acute (< 6 weeks) or subacute LBP (6–12 weeks), provided that they have experienced < 3 LBP episodes during previous 12 months

'Low' or 'moderate' levels of physical activity measured by the IPAQ (< 600 MET-minutes/week)	Unstable angina/uncontrolled cardiac dysrhythmias/severe aortic stenosis/acute systemic infection accompanied by fever

	Medico-legal issues

	Pregnancy

### Randomisation

Patients who have consented to participate will be randomly allocated in accordance with recognised procedures, by computer-generated random allocation sequences that will be prepared centrally by the trial statistician. This sequence will be used to randomly allocate each consenting, numbered subject to one of three study groups: supervised exercise class, walking programme or usual physiotherapy.

Stratification will be by hospital only and separate randomisation lists will be used for each hospital. Prior to randomisation each subject's group allocation preference will be sought and recorded by the Trial Co-ordinator in order to investigate whether treatment preference has any influence on outcomes.

The Trial Co-ordinator will telephone/text the Research Administrator who will provide a central, remote, telephone randomisation service, to obtain each consecutive subject's random group allocation. Following the baseline assessment an appointment for the relevant intervention will then be made by the Trial Co-ordinator. Each participant will receive a copy of 'The Back Book'[38] and be advised to read it before the first physiotherapy appointment. Participants allocated to the WP will be given an educational walking manual, and a Yamax Digiwalker Pedometer, instructed in its use and requested to wear it for seven days to record number of steps and habitual daily activity levels (frequency of walks, walk duration) in an exercise diary prior to the start of the intervention.

### Blinding

#### Clinicians

Due to the nature of the interventions it will not be possible to blind the participating clinicians to the group allocation of patients, but clinicians will not be involved in outcome assessment.

#### Patients

Due to the nature of the interventions it will not be possible to blind patients to their group allocation. The patient information leaflet will inform participants that they have an equal chance of receiving one of three physiotherapy approaches for low back pain management.

#### Assessors

A blinded researcher will administer all outcome measures for postal follow-up. The Trial Co-ordinator will not be involved in follow-up outcome assessment. The statistician will be unaware of group allocation until completion of data analyses.

### Sample Size

A total of 189 patients (n = 63 per group) will be required for 80% power to detect a 4 point difference between groups on the primary outcome, the Oswestry Disability Index [39], with 95% confidence using a two-tail between-within repeated measures ANOVA test of difference between group means. Based on the results of previous LBP trials investigating exercise [11] and using postal follow-ups [40], we anticipate a 30% loss to follow-up. Thus we aim to recruit 82 subjects per group (total 246 subjects).

### Outcome Measures

At the baseline assessment sociodemographic data (i.e. age, gender, education level, social status, occupation and work status, past medical history, LBP history), cardiorespiratory fitness using the shuttle walk test [41], blood pressure, Body Mass Index (BMI, kg/m^2^) and any previous treatment will be documented by the Trial Coordinator. A combination of recommended self-report valid and reliable outcome questionnaires [42] detailed below, and objective measurement will be used at baseline and follow-up (3, 6 and 12 months). The satisfaction questionnaire will only be administered at the end of scheduled intervention period (3 months).

### Clinical Outcomes

The **primary outcome measure **will be a change in:

(i) *Functional disability *due to LBP measured by the Oswestry Disability Index (ODI) [43]

The **secondary outcome measures **will be changes in:

(ii) *Pain *using Numerical Rating Scales for current and worst pain [44]

(iii) *Health-related quality of life *measured by the EuroQol questionnaire [45]

(iv) *Psycho-social beliefs *using the Fear Avoidance Beliefs Questionnaire [46] and the Back Beliefs Questionnaire [47]

(v) *Employment status and number of days reported sick leave over the past year *for those in paid employment only

(vi) *Self-report physical activity levels *using the International Physical Activity Questionnaire (IPAQ) [48],

(vii) *Objective physical activity levels *using an activPal™ device: a valid and reliable measure of walking that is capable of recording steps, time spent lying/sitting, standing and stepping under free living conditions [49] previously used by some of the authors in another LBP trial [50]. A sample of convenience of participants from each group will be requested to wear the activPAL™ on 3 separate occasions each lasting 7 days (1) at baseline, (2) 3 months and (3) 6 months after initial randomisation.

(viii) Self efficacy questionnaire [51]

(ix) Readiness to change questionnaire [52]

(x) *Patient satisfaction *will be assessed using Likert scales assessing satisfaction with outcome and satisfaction with care at 3 months [53]

### Cost Outcomes

Two cost outcomes will be used

(i) *Participants resource utilization *will be assessed using cost diaries developed and successfully used by the Principal Investigator in a previous RCT [54]. A random sample of 20% of cost diaries in each group will be verified by telephone interview with a researcher not involved in the day to day running of the trial to check the accuracy and ensure the quality of the data.

(ii) *Participant's health state *will be assessed using the EuroQol (EQ-5D) Questionnaire's Weighted Health Index [45] and Visual Analogue Scale.

Follow-up reminders will be given by phone and completed questionnaires returned by pre-paid envelopes.

### Interventions

#### (i) Supervised exercise class (SEC)

Within one week of randomisation, participants will commence the SEC. This class will follow a group-based format based on the 'Back to Fitness' programme used in the UK BEAM trial [9-11,13,14] which is underpinned by cognitive behavioural therapy principles designed to change participants behaviour by modifying their attitude to their LBP, i.e. 'hurt' does not mean harm [7,8]. First, each participant will attend the physiotherapy department for an initial individual assessment with the Chartered Physiotherapist delivering the class, where there will be discussion and agreement between the therapist and the patient on short and long-term goals; recording of the patient's exercise capabilities and perceived barriers to recovery and the individual's treatment expectations. Second, participants will attend the physiotherapy department of the relevant participating hospital once a week for 8 weeks for a one-hour supervised group exercise class led by a Chartered Physiotherapist. The physiotherapist will advise patients according to their individual goals and exercise capabilities, and help identify which exercise(s) they could continue independently of the treatment sessions, i.e. foster the development of self-management strategies. Subjects will also be required to rate their perceived exertion during the class on the Borg scale – a linear scale measuring level of breathlessness from 0= 'not breathless at all' to 10 = 'maximal'. [55,56]. Patients will be encouraged to accept responsibility for determining and carrying out their weekly programme of activity. Adherence with the supervised exercise programme will be recorded as the number of sessions attended. The number of sessions defined as adherence will be decided on completion of the trial.

#### (ii) Walking programme (WP)

Within one week of randomisation, participants will commence the WP, the focus being to increase physical activity through a graded walking programme. The WP is based on previous effective programmes in healthy sedentary adults [29,30,57,58] as no previous programmes for LBP are available.

As with the SEC, each participant will attend the physiotherapy department for an initial individual assessment, where there will be discussion and agreement between the therapist and the patient on short and long-term goals; recording of the patient's exercise capabilities and perceived barriers to recovery and the individual's treatment expectations. The therapist will use the information recorded in the exercise diary to inform the starting point for the eight week progressive WP; the minimum being a 10 minute walk (approx 1200 steps) on at least four days per week to be decided with, where possible, one day's rest between walks. The aim of the programme is to progress to the American College of Sports Medicine guidelines of 30 minutes moderate intensity walking on five days per week by week five [20], and then to maintain this level for the remainder of the programme. The 30 minutes brisk walking may be accumulated in two or three shorter bouts if this is more attainable e.g. three 10 minute walks [30,58]. A recent review found no difference in the positive effects on cardiovascular fitness of empirical studies of accumulated or continuous physical activity in sedentary adults and highlighted the need for research to evaluate if accumulated exercise may increase compliance in previously sedentary adults [59]. All participants will be encouraged to use the Borg Breathlessness scale to establish their walking speed: targeting level three (moderate breathlessness) to four (somewhat severe), the minimum level required to achieve the benefits related to exercise [55,56].

Participants will be contacted once per week by telephone by the Chartered Physiotherapist who performed the initial assessment to progress their walking frequency and duration based on their exercise diary record of the previous week's walking, and to provide encouragement. These telephone calls will be based around a specifically developed telephone script based upon CBT principles. Participants will be advised that just like athletes, any unaccustomed exercise is likely to produce some muscle soreness [8]. Participants will use their Yamax Digiwalker Pedometer [60] as a motivational feedback tool, providing immediate information on activity levels [61].

Adherence with the walking programme will be assessed by the frequency, distance, number of steps taken and duration of walks recorded in the exercise diary. Specific adherence levels will be established once the trial is complete. At the end of the intervention subjects will reattend the physiotherapy department for a review appointment with a view to discharge from physiotherapy.

#### (iii) Usual Physiotherapy (UP) -Control Group

Within one week of randomization, participants will commence individual usual physiotherapy at the discretion of the treating physiotherapist in the participating hospital. All physiotherapy treatments and the number of visits will be recorded for the study period in previously designed treatment record forms. On the basis of a previous RCT by the Principal Investigator in the Republic of Ireland public physiotherapy health service the anticipated mean (SD) number of treatments is 5.8 over a mean (SD) of 7.7 weeks (5.8) weeks [54]. A multimodal approach of education/advice, manipulative therapy and exercise therapy will be permitted on the basis of the results of previous surveys of physiotherapy practice in the UK and Ireland [32-34]. As part of this it is expected that subjects will be provided with an individualized exercise programme at the discretion of the treating therapist but will not be permitted to attend group exercise class or undertake a walking programme during the trial. Adherence will be assessed by the number of visits prior to discharge from physiotherapy.

### Adverse effects or events

No adverse events, apart from minor musculoskeletal complaints in the WP group, are anticipated but will be documented by type, length of time, and frequency should they occur [62].

### Data Analysis

Analysis will be by intention to treat. All participants and group allocation will be coded, sociodemographic data coded, and outcome questionnaires scored, and all data entered into the Statistical Package for the Social Sciences database for analysis following data cleaning and checking for errors. Analysis of the clinical outcome data will be performed by the statistician who will remain blinded to group identification until analysis is complete. Between group analyses will be performed to test for between group differences in each outcome from baseline at follow-up. Continuous outcome variables will be analysed using a mixed between-within repeated measures analysis of variance if the assumptions for parametric statistics hold. If substantial evidence of non-normality is found appropriate alternative (non-parametric) tests will be applied, and specified as the primary analysis method.

Using data from the cost diaries, the extent to which each of the interventions has impacted on subsequent use of health care will also be calculated and will cover all identifiable health care costs. The direct and indirect costs to patients receiving each intervention will be obtained. The health care services costs incurred during the intervention and year to follow up will be calculated using the relevant observable prices. Where prices cannot be observed shadow prices will estimated; mean and median health care utilization rates and associated direct service costs in addition to indirect and direct patient costs will be calculated for both intervention groups. Relationships between presenting LBP severity and utilization/cost will be investigated. An additional cost effectiveness analysis will use the magnitude of a mean and median change on the Oswestry Disability Questionnaire as the natural unit of outcome.

The resulting scores from the EQ-5D health state and the visual analog scale will be used to generate the health state utility values necessary for the within trial cost utility analysis (CUA). The CUA will allow the outcomes of this study to be compared to other studies allowing the relevant short term benefit of the interventions to be assessed.

### Qualitative study

A sample of participants from each group will be invited to participate in a semistructured telephone interview or to attend a focus group (one per intervention) at the end of the 6 month follow-up. These subjects will be contacted by letter and invited to contact the Trial Co-ordinator if they wish to participate. Both the focus group and telephone interviews will be conducted by an experienced interviewer with a predetermined set of questions. A "clue and process" format using a checklist of topics, will be used to ensure that the same basic areas are covered but allowing any issues of importance to the participants to emerge. The sessions will be audiotaped, minuted and transcribed verbatim for independent analysis of emergent themes. The main areas to be explored will be subjects' reasons for participation in the trial, their interpretation of study information and documentation, their experiences, expectations and satisfaction with the programme of care including barriers/motivators to participation in the relevant programme.

#### Data Analysis

Qualitiative data from the telephone interviews and focus groups will be analysed using Burnard's thematic analysis [63]. Emerging themes will be identified and comparisons explored between patients' experience of trial participation, their perception of treatment effectiveness and response to each intervention, its impact, motivators and barriers to adherence, as well as their expectations and treatment preferences. A random sample of transcripts from each group will be selected and reviewed by an independent researcher not otherwise involved in the study for inter-rater and intra-rater reliability of identified themes.

## Discussion

We have presented the rationale and design of a randomised controlled trial, with embedded qualitative and economic studies, to investigate and evaluate the difference in effectiveness of a walking programme and a supervised general exercise programme compared to usual physiotherapy, which will act as the control intervention, in subjects with chronic low back pain. The results of this study will be presented as soon as they are available.

## Abbreviations

The following abbreviations have been used in the manuscript: LBP: low back pain; CLBP: chronic low back pain; RCT: randomized controlled trial; CBT: cognitive behavioural therapy; UK: United Kingdom; SEC: supervised exercise class; WP: walking programme.

## Competing interests

The authors declare that they have no competing interests.

## Authors' contributions

All authors were involved in the design of the study. DHO will act as Principal Investigator and was responsible for drafting the paper, and all authors commented on the draft. All authors have read and approved the final manuscript.

## Pre-publication history

The pre-publication history for this paper can be accessed here:


